# A cluster-based randomized controlled trial promoting community participation in arsenic mitigation efforts in Bangladesh

**DOI:** 10.1186/1476-069X-11-41

**Published:** 2012-06-19

**Authors:** Christine Marie George, Alexander van Geen, Vesna Slavkovich, Ashit Singha, Diane Levy, Tariqul Islam, Kazi Matin Ahmed, Joyce Moon-Howard, Alessandro Tarozzi, Xinhua Liu, Pam Factor-Litvak, Joseph Graziano

**Affiliations:** 1 Department of International Health, Program in Global Disease Epidemiology and Control, Johns Hopkins Bloomberg School of Public, 615 N. Wolfe Street, Room E5535, Baltimore, Maryland 21205-2103; 2 Lamont-Doherty Earth Observatory of Columbia University, Palisades, New York, USA; 3 Christian Commission for Development Bangladesh (CCDB), Dhaka, Bangladesh; 4 Department of Biostatistics, Mailman School of Public Health, Columbia University, New York, NY, USA; 5 Columbia University Arsenic & Health Research in Bangladesh, Dhaka, Bangladesh; 6 Department of Geology, Dhaka University, Dhaka, Bangladesh; 7 Department of Sociomedical Sciences, Mailman School of Public Health, Columbia University, New York, NY, USA; 8 Department of Economics, Duke University, Durham, NC, USA; 9 Department of Epidemiology, Mailman School of Public Health, Columbia University, New York, NY, USA

**Keywords:** Arsenic, Health educational intervention, Bangladesh

## Abstract

**Objective:**

To reduce arsenic (As) exposure, we evaluated the effectiveness of training community members to perform water arsenic (WAs) testing and provide As education compared to sending representatives from outside communities to conduct these tasks.

**Methods:**

We conducted a cluster based randomized controlled trial of 20 villages in Singair, Bangladesh. Fifty eligible respondents were randomly selected in each village. In 10 villages, a community member provided As education and WAs testing. In a second set of 10 villages an outside representative performed these tasks.

**Results:**

Overall, 53% of respondents using As contaminated wells, relative to the Bangladesh As standard of 50 μg/L, at baseline switched after receiving the intervention. Further, when there was less than 60% arsenic contaminated wells in a village, the classification used by the Bangladeshi and UNICEF, 74% of study households in the community tester villages, and 72% of households in the outside tester villages reported switching to an As safe drinking water source . Switching was more common in the outside-tester (63%) versus community-tester villages (44%). However, after adjusting for the availability of arsenic safe drinking water sources, well switching did not differ significantly by type of As tester (Odds ratio =0.86[95% confidence interval 0.42-1.77). At follow-up, among those using As contaminated wells who switched to safe wells, average urinary As concentrations significantly decreased.

**Conclusion:**

The overall intervention was effective in reducing As exposure provided there were As-safe drinking water sources available. However, there was not a significant difference observed in the ability of the community and outside testers to encourage study households to use As-safe water sources. The findings of this study suggest that As education and WAs testing programs provided by As testers, irrespective of their residence, could be used as an effective, low cost approach to reduce As exposure in many As-affected areas of Bangladesh.

## Introduction

Exposure to elevated levels of inorganic arsenic (As) is associated with cancers of the skin, bladder, and lung [1-3], developmental effects [4,5], cardiovascular disease [6,7], and skin lesions [8,9]. Chronic As exposure is also associated with deficits in childhood cognitive and motor function [5,10,11]. Recent data suggest associations between chronic As exposure from drinking water and mortality [12].

Groundwater pumped from approximately half the estimated 10 million tubewells in Bangladesh do not meet the World Health Organization (WHO) guideline for As of 10 μg/L [13]. In 2006, Ahmed et al reported that 57% of the estimated population of 28–35 million initially exposed to As above the Bangladesh standard of 50 μg/L remain exposed. The most commonly used As mitigation option is well switching (67%), followed by the use of deep tubewells (28%) [13]. Mitigation options such as piped water systems, rainwater collection, dugwells, As filters, and pond sand filters are utilized by a very small proportion of the population [13,14].

Even when provided with As education, households do not always seek As-safe drinking water sources [15-18]. Testing programs typically involve a representative from an outside organization coming into a village to test the well water for As. These staff label the spout of each well red if the As concentration is greater than 50 μg/L (As–contaminated well) and green if the As concentration in the well is less than 50 μg/L (As-safe well). After the results of the As test are provided, the representative typically leaves the village without providing the resources or in-depth knowledge to address health concerns or mitigation options [15]. The lack of resources at the local level, we hypothesize, may be an important factor limiting the impact of As testing programs. Previous interventions have found that the provision of As education and water arsenic (WAs) testing can encourage households with As contaminated wells to switch to alternative drinking water sources [14,19-21]. However, no studies to date evaluated the effectiveness of having a community member, rather than an outside representative, provide these services.

In 2010, we developed an As education and WAs testing intervention for rural villages in Singair, Bangladesh. Our study objective was to evaluate the effectiveness of having community members, compared to outside representatives, conduct WAs testing and As education. The primary study outcome was switching to an As safe well among those with As contaminated wells at baseline; the secondary outcome was the change in urinary As (UAs) concentration. We hypothesized that the community tester would be more effective since they could provide additional reinforcement by living in the village. Community involvement in As testing may provide a sustainable and less costly option for communities to monitor their As exposure and may represent a model for government or non-governmental agencies to conduct future interventions.

## Methods

### Setting

This study was conducted in rural villages in Singair Upazila, located in the Manikganj district of Bangladesh. This study area was selected due to its wide range of WAs concentrations, and the presence of the Christian Commission for Development Bangladesh (CCDB), a non-governmental organization that assisted with the implementation of this intervention.

### Design

#### Eligibility and Enrollment

We first administered a household drinking water survey to the person responsible for primary drinking water collection in 6746 households in 26 villages [22]. Information was collected about: As status of the household’s primary drinking water source (safe, contaminated, untested), well depth, and well installation date.

**Figure 1:**
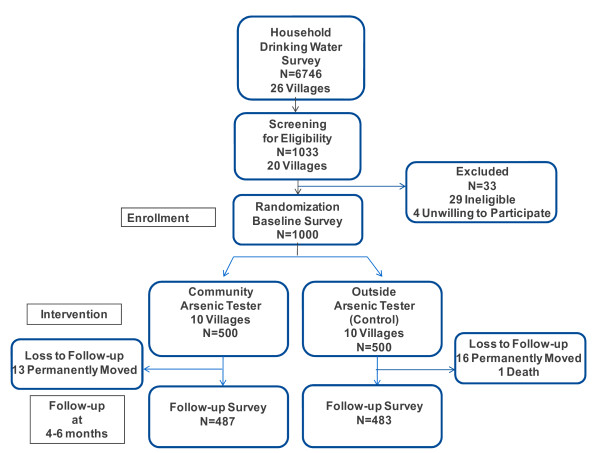
Cluster based randomized controlled trial study design.

Of the 26 villages, 20 met our criteria of having at least 40% of wells exceeding the Bangladesh As standard (50 μg/L), and at least 50 individuals who met the study eligibility criteria (Figure 1). Participants had to: 1) be the person in the household responsible for primary drinking water collection; 2) be using an untested well; and 3) be 18 years of age or older. Villagers were excluded if: 1) they had an As filter; 2) obtained water from an As treatment plant; or 3) did not have a primary well they used to collect the majority of their household’s drinking water (The respondent could be using any well. They did not have to be using a well that they owned.) After confirming the identity and eligibility of participants the interviewer explained the details of the study and obtained informed consent.

This study was a cluster based, randomized controlled trial of 1000 households. Randomization was performed at the village level; participants were clustered within each village. Fifty eligible households were randomly selected based on the household drinking water survey. Each respondent was interviewed at baseline and at follow-up 7–9 months later (Figure 1). In ten villages, a trained community member conducted well WAs testing and provided As education. In the remaining 10 villages, an outside representative, defined as someone living in a different union, performed these tasks. The two groups of villages were geographically separated. Using census data from the Bangladesh Bureau of Statistics, villages were matched on literacy rate and land ownership as these are strong indicators of socioeconomic status [23]. We also attempted to match villages on the proportion of As contaminated wells based on our household drinking water survey. Villages were randomly assigned by the study coordinator to each intervention group at baseline using the random number generator in SAS, version 9.2 (SAS Institute Inc., Cary, NC, USA). Study households in each village were randomly selected in the same manner.

### Intervention

The 10 “community-testers” were forum workers for CCDB who organize community activities on health and poverty alleviation. All As testers were required to be at least 18 years of age and literate. The distribution of age, educational level, gender, and religion did not differ significantly between the community and outside testers.

All testers received a one week intensive training on how to measure the As content of wells and effectively disseminate As education. The tester went to each study household at least once to: 1) measure the As concentration of the household’s primary drinking water source using an As field testing kit; 2) conduct a structured 40 minute As education session; and 3) provide assistance to participants with As contaminated wells to locate a nearby As-safe drinking water source. These tasks were performed in each study village over a period of 3 months.

The As education materials were developed based on ther current scientific literature regarding the health implications of As exposure, studies assessing the knowledge of As in the population [16,17,24,25], and our As education pilot study. Education sessions focused on key messages regarding the health implications of chronic As exposure, and methods to reduce exposure. The sessions were designed to be interactive by asking participants questions about the topics being covered. If a participant’s primary drinking water source was found to be As contaminated, assistance to locate a nearby As-safe drinking water source was provided. In such cases, participants were asked from which water source they would like to collect their drinking and cooking water. If this water source was found to be As-safe and the well owner agreed, the As tester encouraged the participant to collect all of their drinking and cooking water from this source.

### Data Collection

During the baseline and follow-up surveying periods, interviewers visited each study household to: 1) administer a questionnaire to the person responsible for primary drinking water collection; 2) collect a sample of the primary drinking water source; and 3) collect a urine sample from the study respondent.

Both questionnaires obtained information on water usage, socio-demographics characteristics, and knowledge of As. The participant’s knowledge of As was obtained via a 20 item quiz administered at the baseline survey before the start of the intervention and at the follow-up survey. Participants were queried on how to identify As contaminated wells, safe uses of As contaminated water, and the health implications of chronic As exposure. One point was given for a correct item, and zero points for an incorrect item. Possible quiz scores ranged between zero and 20.

### Arsenic Measurements

Urinary As concentrations collected at baseline and follow-up were used as a biological index of As exposure. Previous studies have found strong correlations between urinary As and drinking WAs concentrations [20,26,27]. Switching from an As–contaminated to a safe well can reduce urinary Asconcentrations to a level that approaches those of individuals who have been consistently relying on safe wells [20]. Urine samples were collected from study respondents in 50 ml acid washed tubes during the baseline and follow-up periods. Urine samples were placed in portable coolers, then frozen at −20 °C at the local laboratory in Dhaka, Bangladesh, and shipped on dry ice to Columbia University. Total urinary As was measured using a Perkin-Elmer AAnalyst 600 graphite furnace system, and adjusted for urinary creatinine (Cr) concentrations according to published methods [28]. Our laboratory is part of a quality control program for total urinary As which is coordinated by the Institut de Santé Publique du Québec (Québec, Canada). During the course of this study, the intraclass correlation coefficient between our laboratory’s values and samples calibrated at the Quebec laboratory was 0.99. The average intra-precision and inter-precision for three control urine samples run daily for this period were 2.6%, and 5.7%, respectively.

WAs field testing was conducted using the Hach EZ As Test Kit (Part No. 2822800) which measures As concentrations in water using a colorimetric scale that ranges from 0–500 μg/L. A 40 minute reaction period was used in these studies rather than the manufacturer recommended 20 minutes because a previous study showed that the increased reaction period reduced inconsistencies in the 50–100 μg/L range [22,29].

WAs measurements conducted using the Hach EZ As test Kit were verified by laboratory analysis at the Geochemistry Research Laboratory at the Lamont Doherty Earth Observatory (LDEO) at Columbia University. The As concentrations were measured using Inductively-Coupled Plasma-Mass Spectrometry (ICP-MS) with a detection limit of 0.1 μg/L [30,31].

### Statistical Methods

The primary hypothesis of this study was that training a community member to perform As testing and provide As education is more effective than sending a trained person from outside the village to conduct these same tasks, conditional on equal competence and similar observed characteristics of the tester.

Based on a previous study conducted in Araihazar, Bangladesh, we assumed that the proportion of well switching would be 0.33 in our outside tester villages and 0.66 in our community-tester villages [14,21]. Furthermore, based on the results of our household drinking water survey we estimated that approximately 50% (500) of the 1000 respondents included into our study population would be using wells that were As contaminated. We specified the type 1 error, the probability of rejecting H_o_ when it is true, at 5% and the type 2 error, the probability of not rejecting H_o_ when it is false, at 20%. Thus, we required 18 villages with 35 households each. To account for at least a 10% loss to follow up, we selected a sample size of 20 villages of 50 households each.

The outcome variables in this study were: 1) questionnaire reported well switching; and 2) change in urinary As concentration. We evaluated the determinants of well switching for study respondents with As–contaminated wells at baseline. Safe and As–contaminated were defined according to the Bangladesh WAs standard of 50 μg/L. Chi-square tests and two sample t-tests were used to compare differences between the community-tester and outside-tester villages for categorical and continuous variables, respectively.

Logistic regression was used to estimate the odds of well switching controlling for both individual and village level covariates. Generalized estimating equations (GEE) were used to account for clustering within villages [32]. We estimated the most parsimonious model by eliminating all non statistically significant variables (p >0.05), except for those *a priori* specified (ie. Type of As Tester) until the lowest quasi likelihood information criterion (QIC) was determined [32]. All analyses were performed using SAS, version 9.2 (SAS Institute Inc., Cary, NC, USA).

### Ethics Section

The study protocol was approved by the Columbia University Medical Center Institutional Review Board and the Bangladesh Medical Research Council. Informed consent was obtained from all study respondents.

## Results

During our baseline survey, 1033 respondents with untested wells, selected from our household drinking water survey, were screened for eligibility. Of these, 4 (0.4%) were unwilling to participate and 29 (2.8%) were ineligible. At follow-up, 30 (3%) respondents had either permanently moved [29] or died [1]. Urine was collected from 953 (95%) respondents at baseline and 930 (96%) respondents at follow-up. Five hundred and forty three (56%) respondents were found to be using As contaminated wells, and 427 (44%) were found to be using As safe wells.

The distribution of age, literacy, religion, baseline quiz score, and land ownership did not differ significantly between the two intervention groups. However, the community tester intervention group had more well owners, more As contaminated wells, and lived further away from an As-safe well; they also had significantly higher urinary As concentrations at baseline (Table 1). The number of times the participant met with the As tester was significantly higher for the community-tester versus outside-tester villages; 48% of participants in the community-tester villages met with the As tester four or more times, compared to 13% in the outside-tester villages.

**Table 1:** Baseline and follow-up characteristics by arsenic tester village

**Characteristics**	**Community tester villages (N = 487)**	**Outside tester villages (N = 483)**	**P-value**
**Age (yrs) [**Mean ± SD(Range)]	36.3 ± 11.4 (18–102)	37.8 ± 12.8(18–86)	0.07
**Gender (%)**			
Female	99.8	100	0.32
**Religion (%)**			
Muslim	93	95	0.14
Hindu	7	5	
**Respondent can read and write (%)**			
Yes	42	40	0.54
**Head of household education (%)**			
No education	52	55	0.23
**Respondent baseline knowledge of arsenic quiz score mean ± SD(range)**	8.5 ± 3.0(0–18)	8.4 ±2.9(0–17)	0.77
**Radio ownership (%)**			
Yes	25	28	0.36
**Land ownership (%)**			
No land ownership	12	18	0.07
Less than 1 Acre	63	57	
1 to 2 Acres	25	25	
**Well ownership (%)**			
Yes	82	75	0.01
**Proportion of unsafe wells in respondent's village(%)**			
0-60 %	30	68	<.0001
**Minutes to an arsenic safe drinking water source for unsafe well owners (%) (N = 587)**			
Less than or equal to 5 minutes	68	32	<.0001
**Arsenic status of tubewell**			
Safe	39	49	0.004
**Baseline water arsenic [μg/L(Mean ± SD(range))]**^1^	124 ± 145 (0–500)	117 ± 147 (0–500)	0.66
**Baseline creatinine-adjusted urinary As [μg/g Cr (Mean ± SD(range))]**^1^	178 ± 122.0(9–901)	143 ±132(18–1060)	0.0002

*P-values were calculated using a chi-square test for categorical variables and a 2 sample t-test for continuous variables.

1. Water and Urinary Arsenic were log-transformed.

**Table 2:** Predictors of well switching among unsafe well users

	**Total**^**1**^	**% Who switched**^**2**^	**OR for switching (95% CI)**^**3**^
**Arsenic tester**			
Outside arsenic tester	248	63	1.00
Community arsenic tester	295	44	0.86 (0.42-1.77)
**Proportion of unsafe wells in respondent's village**			
Less than 60%	258	72	1.00
Greater or equal to 60%	285	35	0.25 (0.13-0.48)
**Minutes to safe drinking water source**			
Less than or equal to 5 minutes	282	63	1.00
Greater than 5 minutes	227	43	0.55 (0.32-0.96)
**Well ownership**			
No	103	67	1.00
Yes	440	50	0.41 (0.25-0.65)
**Radio ownership**			
No	398	55	1.00
Yes	145	47	0.64 (0.43-0.94)

(1) "Total" indicates the number of respondents with each attribute. (2) "% Switching" indicates the percentage of individuals with that attribute that switched wells. (3) OR were adjusted for all variables in the table. Participants with unknown information for any of the covariates were excluded.

Overall, 53% of respondents with As contaminated wells at baseline switched during the intervention period. Switching was more common in the outside-tester (63%) versus community-tester villages (44%). However, after adjusting for the availability of As safe drinking water sources, the association between the As tester and well switching was not significant (OR =0.86[95% CI 0.42-1.77) (Table 3). Follow-up knowledge of As quiz scores were positively related to well switching, although the association did not reach statistical significance (Table 4). The number of times the participants met with an As tester was positively associated with well switching, when the As tester met with the study respondent at least 4 times (OR = 1.61; 95% (1.11 - 2.35)).

**Table 3:** Predictors of well switching among unsafe well users

	**Total**^**1**^	**% Who switched**^**2**^	**OR for switching (95% CI)**^**3**^
**Follow-up knowledge of arsenic quiz score**			
Q1 (0–11)	102	50	1.00
Q2 (12–14)	146	43	0.76 (0.52-1.12)
Q3 (15–16)	103	57	1.22 (0.71-2.10)
Q4 (17–20)	192	59	1.26 (0.86-1.85)
**Number of times met with arsenic tester**			
1 Time	154	53	1.00
2 Times	138	52	1.24 (0.82-1.86)
3 Times	85	52	1.24 (0.80-1.93)
4 or more times	166	54	1.61 (1.11-2.35)

(1) "Total" indicates the number of respondents with each attribute. (2) "% Switching" indicates the percentage of individuals with that attribute that switched wells. (3) ORs are *unadjusted*. Participants with unknown information for any of the covariates were excluded.

**Table 4:** Follow-up characteristics by arsenic tester village

**Characteristics**	**Community tester villages (N = 487)**	**Outside tester villages (N = 483)**	**P-value**
**Respondent follow-up arsenic knowledge quiz score**	14.3 ± 3.2(4–20)	14.0 ± 3.6(4–20)	0.2447
**Number of times met with arsenic tester(%)**			
1 Time	23	29	<0.0001
2 Times	18	36	
3 Times	11	22	
4 or more times	48	13	
**Switching status (%)**			
Did Not Switch	56	37	<.0001
Switched	44	63	
**Reason for switching, amoung those unsafe well users who switched(%) (N = 287)**			
Previous tubewell was unsafe for arsenic	87	95	0.121
Previous tubewell broken	4	<1	
Too many people using previous tubewell	<1	<1	
Dug a new tubewell	4	<1	
Did not like the taste of previous tubewell	2	<1	
Did not like the color of previous tubewell	2	1	
None of these	<1	1	
**Reason for not switching, amoung those unsafe well users who did not switched(%) (N = 256)**			
Distance of the safe tubewell was too far	54	58	0.087
Family owns its own tubewell and doesn't wish to impose on others	15	23	
Arsenic safe well had too many users	5	2	
Safe well owner near home does not want to share	14	9	
Physical Limitation	5	2	
Alternative well had bad taste	3	1	
Alternative well had unusual color	1	2	
None of these	1	4	
**Follow-up creatinine-adjusted urinary As [μg/g Cr (Mean ± SD(range))]**	163 ±157(17–1241)	128 ± 150(24–1905)	<.0001
**Number of arsenic test conducted**	835	675	0.0069

*P-values were calculated using a chi-square test for categorical variables and a 2 sample t-test for continuous variables 1. Urinary Arsenic were log-transformed.

Participants who lived in villages with > 60% As contaminated wells, classification used by the Bangladeshi and UNICEF, were significantly less likely to switch in comparison to those who lived in villages with < 60% As–contaminated wells (OR = 0.25; 95% CI (0.13-0.48)). In villages with less than 60% As–contaminated wells, 74% of study households in the community tester villages, and 72% of households in the outside tester villages reported switching to an As safe drinking water source. In contrast to only 35% wells switching in villages with greater than 60% As contaminated wells. In addition, participants who required more than 5 minutes to walk to an As-safe drinking water source were significantly less likely to switch in comparison to those who lived within 5 minutes of an As-safe drinking water source (OR = 0.55; 95% CI [0.32-0.96]). Finally, participants who owned their own well were significantly less likely to switch in comparison to those who did not own their own well (OR = 0.41; 95% CI (0.25-0.65)).

Among participants with As contaminated wells who changed their drinking water source, the most common reported reason for switching was that their baseline well was As–contaminated (92%). The most common reported reasons for *not* switching wells were: 1) long distance to a safe well (57%); 2) family ownership of well (20%); and 3) owner(s) of safe wells near the respondent’s home do not want to share (11%). Eight percent of respondents with *safe* wells at baseline switched. The most common reported reason for well switching among these respondents were: 1) did not like the taste of their previous well water (23%); 2) dug a new well (17%); and 3) previous well broke (17%). Similar reasons were given by participants in the two intervention groups.

**Figure 2:**
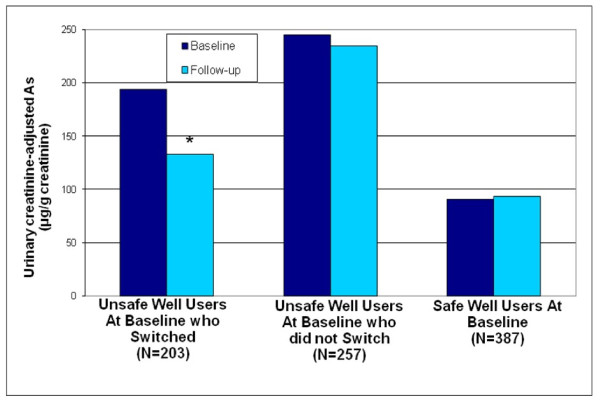
Mean urinary creatinine-adjusted As levels for study respondents *P < .0001 as compared to baseline using a paired t-test where urinary arsenic was log transformed.

Overall baseline mean urinary As concentrations were more than double among respondents with As contaminated wells (215 μg As /g Cr) as compared to those using safe wells (91 μg As /g Cr). At follow-up, the overall mean urinary As concentrations for those with As contaminated wells who switched to safe wells decreased significantly from 194 to 133 μg As/g Cr (Figure 2); the reduction did not differ between intervention groups. UAs was essentially the same for those who used As contaminated wells at baseline but did not switch wells (245 vs 234 μg As /g Cr). Finally, there was no appreciable change in urinary As concentrations for safe well users. There were a significantly higher number of As tests conducted in the community tester (835) versus outside tester villages(675). This is likely due to the higher number of As contaminated wells in the community testing villages, resulting in the need for additional As testing to locate As safe drinking water sources.

## Discussion

Millions in Bangladesh continue to drink groundwater containing elevated levels of As [13]. Many households lack access to As testing services, preventing them from knowing the As status of their wells and locating As-safe water sources in their villages. Thus, there is an urgent need for effective As education and WAs testing programs in Bangladesh [13-15]. This study is the first randomized trial evaluating the effectiveness of community participation in As mitigation in Bangladesh. We hypothesized that community-testers would be more effective than outside-testers in terms of reducing As exposure because the former would offer additional reinforcement by living within the community. Although our data did not support this hypothesis, the intervention program was very successful in encouraging households to use As-safe drinking water sources. Fifty-three percent of participants with As contaminated wells at baseline switched wells at follow-up, mostly because their baseline well was As contaminated relative to As.

We observed that the reinforcement provided by the availability of an As tester within the village was positively related to well switching. Through their continued presence, the community-tester provided significantly more reinforcement in the village than the outside-tester as evidenced by the number of contacts between the participants and the testers. The knowledge of As quiz scores were significantly higher for respondents at follow-up, compared to baseline, for both intervention groups (Unpublished).

We observed significant reductions in UAs concentrations for As–contaminated well users who reported switching wells at follow-up, indicating that our intervention was successful in reducing a biomarker of As exposure. Previous studies in Taiwan indicate that a reduction of As exposure may reduce associated mortality from renal diseases [33,34], intracerebral hemorrhage [35], and ischemic heart disease [36]. A study in Chile found that reduced As intake was associated with decreased numbers of micro-nucleated cells in the bladder [37].

Sources of dietary As such as cooking rice in contaminated water or using rice with elevated As concentration can contribute to elevated urinary As concentrations [38,39]. Therefore it is possible that food As has contributed to the ingested dose of As in our study population. However the literature suggests that when water As concentrations exceed 50 μg/L that ingested water As is the dominant exposure route [39]. In our educational program we encouraged households to both drink and cook with As safe water. Only 8% of study respondents using As contaminated wells at baseline who switched to safe wells at follow-up reported using their previous tubewell for cooking.

Our findings are consistent with an intervention for 11,746 participants conducted in Araihazar, Bangladesh. That intervention, administered over a two-year period, involved WAs testing and labeling, village level As education, and the targeted installation of deep tubewells with low WAs. At follow-up, 58% of As contaminated well users and 17% of safe well users had switched to new drinking water sources. A 46% reduction in UAs was observed for those with As–contaminated wells who switched to As-safe drinking water sources [20]. Our current intervention was conducted over a much shorter duration and did not involve the installation of deep tubewells, yet we observed roughly comparable results.

The unavailability of As-safe drinking water sources, i.e. the proportion of As–contaminated wells, in a village was the greatest barrier to well switching. In villages with less than 60% As contaminated wells, 72% of respondents with As–contaminated wells switched, compared to 35% well switching in villages with greater or equal to 60% As–contaminated wells. This is consistent with Hanchett et al., who found that the unavailability of As-safe water sources was a barrier to well switching in six districts of Bangladesh [15]. In our study, the time to walk to As–safe water source was also a significant barrier to well switching. Previous studies have indicated that well switching significantly declines if the nearest safe well is located more than 100 meters away [14,20,21]. Well ownership was also a significant barrier to well switching, likely because well owners are more reluctant to shift from a well in which they invested their own money. All of these barriers to well switching were significantly higher in the community versus outside tester villages suggesting a possible reason for the lower well switching observed in these villages.

Our study suggests that WAs testing and As education programs would be most effective in areas where <60% of wells are As-contaminated. In these villages the vast majority of respondents with contaminated wells switched (72%). A recent report of a nationwide survey in Bangladesh indicated that 77% of the population lives in areas with between 0-60% of their wells being As contamination [40]. Therefore our intervention is a viable option for the majority of the population residing in As affected areas of Bangladesh. For the 23% of the population who reside in areas with > 60% As–contaminated wells, this intervention will likely need to be combined with the provision of alternative mitigation options such as the installation of deep tubewells, As filters, or rain water harvesting.

A major limitation of our study was the relatively short three month duration of our intervention period. We hypothesized that community-testers would be more effective than outside-testers because of their additional reinforcement. While we did observe that the community-testers provided significantly more reinforcement than the outside testers, this did not appear to increase their effectiveness in reducing As exposure. We attribute this result in part to the significantly higher proportion of As–contaminated well located in the community-tester villages and in part to the short duration of the study. Nevertheless, the use of the community-testers provides a potentially sustainable approach for As mitigation because of the continued presence of the testers in villages over time to provide additional reinforcement and WAs testing services. Further, community testers will likely be less costly because they do not require transportation costs. We recommend that if this intervention approach is upscaled that it be incorporated in existing community health worker programs conducted by non-governmental organizations or by local government. This would reduce the required operation cost. Working with existing organizations would also allow for greater accountability of those providing the As testing and education and make refresher trainings over time easier to organize.

## Conclusions

In conclusion, the overall intervention was effective in reducing As exposure provided there were As-safe drinking water sources available. However, there was not a significant difference observed in the ability of the community and outside testers to encourage study households to use As-safe water sources. The findings of this study suggest that As education and WAs testing programs provided by As testers, irrespective of their residence, could be used as an effective, low cost, easy to deliver intervention approach to reduce As exposure in many As-affected areas of Bangladesh. Furthermore, this approach has the advantage of not involving costly As filters, deep tubewells, or As treatment plants.

## Competing Interests

The authors have no competing interests.

## Authors’ contributions

This study was a multidisciplinary international collaboration that required significant expertise of scientists with diverse public health, earth sciences, and social science. For this reason there are twelve authors on the paper. C M G directed the field study, performed the statistical analysis, and wrote the first draft of the manuscript. J G and A V G directed the studies and revised the manuscript. Pam Factor-Litvak assisted with the analysis of the data presented in the manuscript and provided substantial comments to several drafts. All individuals named on the article provided comments to several drafts of the article and approved the final version.
